# Countably *QC*-Approximating Posets

**DOI:** 10.1155/2014/123762

**Published:** 2014-08-05

**Authors:** Xuxin Mao, Luoshan Xu

**Affiliations:** ^1^College of Science, Nanjing University of Aeronautics and Astronautics, Nanjing 210016, China; ^2^Department of Mathematics, Yangzhou University, Yangzhou 225002, China

## Abstract

As a generalization of countably *C*-approximating posets, the concept of countably *QC*-approximating posets is introduced. With the countably *QC*-approximating property, some characterizations of generalized completely distributive lattices and generalized countably approximating posets are given. The main results are as follows: (1) a complete lattice is generalized completely distributive if and only if it is countably *QC*-approximating and weakly generalized countably approximating; (2) a poset *L* having countably directed joins is generalized countably approximating if and only if the lattice *σ*_*c*_(*L*)^*op*^ of all *σ*-Scott-closed subsets of *L* is weakly generalized countably approximating.

## 1. Introduction

The notion of continuous lattices as a model for the semantics of programming languages was introduced by Scott in [1]. Later, a more general notion of continuous directed complete partially ordered sets (i.e., continuous dcpos or domains) was introduced and extensively studied (see [2–4]). Lawson in [4] gave a remarkable characterization that a dcpo *L* is continuous if and only if the lattice *σ*(*L*)^*op*^ of all Scott-closed subsets of *L* is completely distributive. Gierz et al. in [5] introduced quasicontinuous domains, the most successful generalizations of continuous domains, and proved that quasicontinuous domains equipped with the Scott topology are precisely the spectra of hypercontinuous distributive lattices. Venugopalan in [6] introduced generalized completely distributive (GCD) lattices and Xu in his Ph.D. thesis [7] proved that GCD lattices are precisely the dual of hypercontinuous lattices. Ho and Zhao in [8] introduced the concept of *C*-continuous lattices. And they showed that for any poset *L*, *σ*(*L*)^*op*^ is a *C*-continuous lattice and that *L* is continuous if and only if *σ*(*L*)^*op*^ is continuous.

On the other hand, Lee in [9] introduced the concept of countably approximating lattices, a generalization of continuous lattices, and showed that this new larger class has many properties in common with continuous lattices. In [10], Han et al. further generalized the concept of countably approximating lattices to the concept of countably approximating posets and characterized countably approximating posets via the *σ*-Scott topology. Yang and Liu in [11] introduced the concept of generalized countably approximating posets, a generalization of countably approximating posets. Making use of the ideas of [8, 10], Mao and Xu in [12] introduced the concept of countably *C*-approximating posets and showed that the lattice of all *σ*-Scott-closed subsets of a poset is a countably *C*-approximating lattice and that a complete lattice is completely distributive if and only if it is countably approximating and countably *C*-approximating.

In this paper, we generalize the concept of countably *C*-approximating posets to the concept of countably *QC*-approximating posets. With the countably *QC*-approximating property, we present some characterizations of GCD lattices and generalized countably approximating posets.

## 2. Preliminaries

We quickly recall some basic notions and results (see, e.g., [3, 8] or [11]). Let (*L*, ≤) be a poset. Then *L* with the dual order is also a poset and denoted by *L* ^*op*^. A* principal ideal* (resp.,* principal filter*) is a set of the form ↓*x* = {*y* ∈ *L*∣*y* ≤ *x*} (resp., ↑*x* = {*y* ∈ *L*∣*x* ≤ *y*}). For *X*⊆*L*, we write ↓*X* = {*y* ∈ *L*∣∃   *x* ∈ *X*, *y* ≤ *x*}, ↑*X* = {*y* ∈ *L*∣∃   *x* ∈ *X*, *x* ≤ *y*}. A subset *X* is *a*(*n*)* lower set* (resp.,* upper set*) if *X* =  ↓*X* (resp., *X* =  ↑*X*). The supremum of *X* is denoted by ∨*X* or sup⁡*X*. A subset *D* of *L* is* directed* if every finite subset of *D* has an upper bound in *D*. A subset *D* of *L* is* countably directed* if every countable subset of *D* has an upper bound in *D*. Clearly every (countably) directed set is nonempty, and every countably directed set is directed but not vice versa. A poset *L* is a* directed complete partially ordered set* (dcpo, in short) if every directed subset of *L* has a supremum. A poset is said to have countably* directed joins* if every countably directed subset has a supremum.

**Remark 1 .**

It is clear that if *D* is countably directed and itself is countable, then *D* has a maximal element. By this observation, we see that every countable poset must have countably directed joins and thus a poset having countably directed joins need not be a dcpo.

The following definitions give various induced relations by the order of a poset.

**Definition 2 (see [3]).**

Let *L* be a poset and *x*, *y* ∈ *L*. We say that *x* is* way-below y* or *x approximates y*, written *x* ≪ *y* if whenever *D* is a directed set that has a supremum sup⁡*D* ≥ *y*, then there is some *d* ∈ *D* with *x* ≤ *d*. For each *x* ∈ *L*, we write ⇓*x* = {*y* ∈ *L*∣*y* ≪ *x*}. A poset is said to be* continuous* if every element is the directed supremum of elements that approximate it. A continuous poset which is also a complete lattice is called a* continuous lattice*.

**Definition 3 (see [10]).**

Let *L* be a poset and *x*, *y* ∈ *L*. We say that *x* is* countably way-below y*, written *x*  ≪_*c*_ *y* if for any countably directed subset *D* of *L* with sup⁡*D* ≥ *y*, there is some *d* ∈ *D* with *x* ≤ *d*. For each *x* ∈ *L*, we write ⇓_*c*_ *x* = {*y* ∈ *L*∣*y* ≪_*c*_ *x*} and ⇑_*c*_ *x* = {*y* ∈ *L*∣*x*  ≪_*c*_ *y*}. A poset *L* having countably directed joins is called a* countably approximating poset* if for each *x* ∈ *L*, the set ⇓_*c*_ *x* is countably directed and *x* = ∨⇓_*c*_ *x*. A countably approximating poset which is also a complete lattice is called a* countably approximating lattice*.

In a poset *L*, it is clear that *x* ≪_*c*_ *y* implies that *x* ≤ *y*. Since every countably directed set is directed, we have that *x* ≪ *y* implies *x* ≪_*c*_ *y* for all *x*, *y* ∈ *L*. In other words, ⇓*y*⊆⇓_*c*_ *y* for each *y* ∈ *L*. However, the following example shows that the reverse implication need not be true.

**Example 4 .**

Let *L* be the unit interval [0,1]. For all *x*, *y* ∈ [0,1], it is easy to check that *x*≪_*c*_ *y*⇔*x* ≤ *y* and that *x* ≪ *y*⇔*x* = 0 = *y* or *x* < *y*.

By Remark 1, it is clear that every countable poset is a countably approximating poset.

**Proposition 5 .**

Let *L* be a poset and *S* a countable subset of *L* such that ∨*S* exists. If *s*≪_*c*_ *x* for all *s* ∈ *S*, then ∨*S*≪_*c*_ *x*.

**Proof**

Straightforward.

By Proposition 5, in a complete lattice *L*, the set ⇓_*c*_ *x* is automatically countably directed for each *x* ∈ *L*. So, a complete lattice *L* is countably approximating if and only if for each *x* ∈ *L*, *x* = ∨⇓_*c*_ *x*. Thus every continuous lattice is a countably approximating lattice.

**Proposition 6 .**

Let *L* be a poset. If every countably directed subset of *L* has a maximal element, then *L* is a countably approximating poset.

**Proof**

Straightforward by Definition 3.

**Example 7 .**

**Figure 1:**
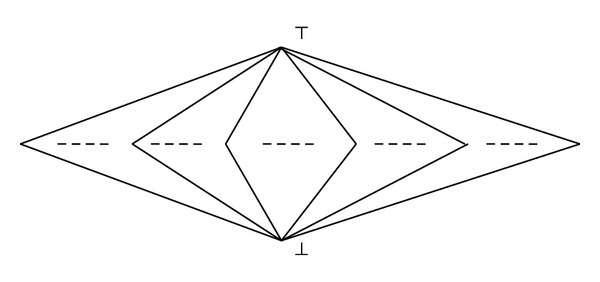
A complete lattice with countably directed sets having maximal elements.

Let *L* be the complete lattice formed by uncountably many incomparable unit intervals [0,1] with all the 0's being pasted as a ⊥ and all the 1's being pasted as a *⊤* (See Figure 1). Then it is easy to check that the resulting complete lattice satisfies the condition in Proposition 6 and thus is a countably approximating lattice.

**Proposition 8 .**

Let *L* be a poset. If every countably directed subset of *L* is countable, then *L* is a countably approximating poset.

**Proof**

It is straightforward by Remark 1 and Proposition 6.

**Example 9 .**

If *N* with its usual order is augmented with uncountably many incomparable upper bounds, then it is easy to check that the resulting poset satisfies the condition in Proposition 8 and thus is a countably approximating poset.

For a set *X*, we use *P*(*X*) to denote the power set of *X* and *P*_fin_(*X*) to denote the set of all nonempty finite subsets of *X*. For a poset *L*, define a preorder ≤ (sometimes called* Smyth preorder*) on *P*(*L*)∖{*∅*} by *G* ≤ *H* if and only if ↑*H*⊆↑*G* for all *G*, *H*⊆*L*. That is, *G* ≤ *H* if and only if for each *y* ∈ *H* there is an element *x* ∈ *G* with *x* ≤ *y*. We say that a nonempty family *F* of subsets of *L* is (countably)* directed* if it is (countably) directed in the Smyth preorder. More precisely, *F* is directed if for all *F*_1_, *F*_2_ ∈ *F*, there exists *F* ∈ *F* such that *F*_1_, *F*_2_ ≤ *F*; that is, *F*⊆↑*F*_1_∩↑*F*_2_.

Generalizing the relation ≪_*c*_ on points of *L* to the nonempty subsets of *L*, one obtains the concept of weakly generalized countably approximating posets.

**Definition 10 .**

Let *L* be a poset having countably directed joins. A binary relation ≪_*c*_ on *P*(*L*)∖{*∅*} is defined as follows. *A*≪_*c*_ *B* if and only if for any countably directed set *D*⊆*L*, ∨  *D* ∈ ↑*B* implies *D* ∩↑*A* ≠ *∅*. We write *F*≪_*c*_ *x* for *F*≪_*c*_{*x*} and *y*≪_*c*_ *H* for {*y*}≪_*c*_ *H*. If for each *x* ∈ *L*, ↑*x* = ∩{↑*F*∣*F* ∈ *ω*(*x*)}, where *ω*(*x*) = {*F*∣*F* ∈ *P*_fin_(*L*) and *F*≪_*c*_ *x*}, then *L* is called a* weakly generalized countably approximating poset*. A weakly generalized countably approximating poset which is also a complete lattice is called a* weakly generalized countably approximating lattice.*

A weakly generalized countably approximating poset (lattice) *L* with the condition that for each *x* ∈ *L*, *ω*(*x*) is countably directed is called a* generalized countably approximating poset* (*lattice*) in [11].

As a generalization of completely distributive lattice, the following concept of GCD lattices was introduced in [6].

**Definition 11 (see [6]).**

Let *L* be a poset. A binary relation ⊲ on *P*(*L*) is defined as follows. *A*⊲*B* if and only if whenever *S* is a subset of *L* for which ∨*S* exists, ∨*S* ∈ ↑*B* implies *S* ∩↑*A* ≠ *∅*. A complete lattice *L* is called a* generalized completely distributive lattice* or shortly a GCD lattice, if and only if for all *x* ∈ *L*, ↑*x* = ∩{↑*F*∣*F* ∈ *P*_fin_(*L*) and *F*⊲*x*}.

**Definition 12 (see [3]).**

A subset *U* of a poset *L* is* Scott-open* if ↑*U* = *U* and for any directed set *D*⊆*L*, sup⁡*D* ∈ *U* implies *U*∩*D* ≠ *∅*. All the Scott-open sets of *L* form a topology, called the* Scott topology* and denoted by *σ*(*L*). The complement of a Scott-open set is called a* Scott-closed set*. The collection of all Scott-closed sets of *L* is denoted by *σ*(*L*)^*op*^. The topology on *L* generated by {*L*∖↓*x*∣*x* ∈ *L*} as a subbase is called the* upper topology* and denoted by *ν*(*L*).

Replacing directed sets with countably directed sets in Definition 12, we can get the concept of *σ*-Scott-open sets.

**Definition 13 (see [10]).**

Let *L* be a poset. A subset *U* of *L* is called *σ*-*Scott-open* if ↑*U* = *U* and for any countably directed set *D*⊆*L*, sup⁡*D* ∈ *U* implies *U*∩*D* ≠ *∅*. All the *σ*-Scott-open sets of *L* form a topology, called the *σ*-*Scott topology* and denoted by *σ*_*c*_(*L*). The complement of a *σ*-Scott-open set is called a *σ*-*Scott-closed* set. The collection of all *σ*-Scott-closed sets of *L* is denoted by *σ*_*c*_(*L*)^*op*^.

**Remark 14 (see [10], Remark 2.1).**

(1) For a poset *L*, the *σ*-Scott topology *σ*_*c*_(*L*) is closed under countably intersections and the Scott topology *σ*(*L*) is coarser than *σ*_*c*_(*L*); that is, *σ*(*L*)⊆*σ*_*c*_(*L*).

(2) A subset of a poset is *σ*-Scott-closed if and only if it is a lower set and closed under countably directed joins.

To study the order structure of the lattice of all *σ*-Scott-closed subsets for a poset, Mao and Xu in [12] introduced the concept of countably *C*-approximating posets.

**Definition 15 (see [12]).**

Let *L* be a poset and *x*, *y* ∈ *L*. We say that *x* is *σ*-*beneath y*, denoted by *x*≺_*σ*_ *y*, if for any nonempty *σ*-Scott-closed set *F*⊆*L* for which ∨*F* exists, ∨*F* ≥ *y* always implies that *x* ∈ *F*. Poset *L* is said to be* countably C-approximating* if for each *x* ∈ *L*, *x* = ∨↓^≺_*σ*_^ *x*, where ↓^≺_*σ*_^ *x* = {*y* ∈ *L*∣*y*≺_*σ*_ *x*}. A complete lattice which is also countably *C*-approximating is called a* countably C-approximating lattice*.

**Lemma 16 (see [12]).**

For a poset *L*, the lattice *σ*_*c*_(*L*)^*op*^ is countably *C*-approximating.

**Proof**

Let *L* be a poset and *C* ∈ *σ*_*c*_(*σ*_*c*_(*L*)^*op*^)^*op*^. It is straightforward to check that ⋁_*σ*_*c*_(*L*)^*op*^_ *C* = ∪*C*. For each *F* ∈ *σ*_*c*_(*L*)^*op*^, we have that *F* = ⋁_*σ*_*c*_(*L*)^*op*^_{↓*x*∣*x* ∈ *F*}. Suppose *C* ∈ *σ*_*c*_(*σ*_*c*_(*L*)^*op*^)^*op*^ with ⋁_*σ*_*c*_(*L*)^*op*^_ *C*⊇*F*. Then for each *x* ∈ *F*, since ⋁_*σ*_*c*_(*L*)^*op*^_ *C* = ∪*C*⊇*F*, there exists *A* ∈ *C* such that *x* ∈ *A*. Noticing that *A* ∈ *σ*_*c*_(*L*)^*op*^ is a lower set, we have ↓*x*⊆*A* ∈ *C*. It follows from *C* ∈ *σ*_*c*_(*σ*_*c*_(*L*)^*op*^)^*op*^ being a lower set that ↓*x* ∈ *C*. Thus by Definition 15, ↓*x*≺_*σ*_ *F* holds in *σ*_*c*_(*L*)^*op*^. Hence, *F* = ⋁_*σ*_*c*_(*L*)^*op*^_{↓*x*∣*x* ∈ *F*}⊆∨↓^≺_*σ*_^ *F*⊆*F*. So, *F* = ∨↓^≺_*σ*_^ *F* and by the arbitrariness of *F* ∈ *σ*_*c*_(*L*)^*op*^, we conclude that *σ*_*c*_(*L*)^*op*^ is countably *C*-approximating.

## 3. Countably *QC*-Approximating Posets

In this section, we introduce the concept of countably *QC*-approximating posets. Firstly, we generalize the relation ≺_*σ*_ on points of a poset *L* to the nonempty subsets of *L*.

**Definition 17 .**

For a poset *L*, the *σ*-beneath relation ≺_*σ*_ on nonempty subsets of *L* is defined as follows: *A*≺_*σ*_ *B* if and only if whenever *S* is a nonempty *σ*-Scott-closed subset of *L* for which ∨*S* exists, ∨*S* ∈ ↑*B* implies *S* ∩↑*A* ≠ *∅*. We write *F*≺_*σ*_ *x* for *F*≺_*σ*_{*x*}. Set *c*(*x*) = {*F*∣*F* ∈ *P*_fin_(*L*)  and  *F*≺_*σ*_ *x*}.

The next proposition is basic and the proof is omitted.

**Proposition 18 .**

Let *L* be a poset. Then

- ∀*G*, *H*⊆*L*, *G*≺_*σ*_ *H*⇒*G* ≤ *H*;
- ∀*G*, *H*⊆*L*, *G*≺_*σ*_ *H*⇔∀*h* ∈ *H*, *G*≺_*σ*_ *h*;
- ∀*E*, *F*, *G*, *H*⊆*L*, *E* ≤ *G*≺_*σ*_ *H* ≤ *F*⇒*E*≺_*σ*_ *F*;
- ∀*x*, *y* ∈ *L*, {*x*}≺_*σ*_{*y*}⇔*x*≺_*σ*_ *y*.

With the relation ≺_*σ*_, we have the concept of countably *QC*-approximating posets.

**Definition 19 .**

A poset *L* is said to be* countably quasi*-*C*-*approximating*, shortly* countably* *QC*-*approximating*, if for all *x* ∈ *L*, ↑*x* = ∩{↑*F*∣*F* ∈ *c*(*x*)}. A countably *QC*-approximating poset which is also a complete lattice is called a* countably* *QC* *-approximating lattice*.

**Proposition 20 .**

Countably *C*-approximating posets are countably *QC*-approximating.

**Proof**

Let *L* be a countably *C*-approximating poset. Then for all *x* ∈ *L*,

(1) ↑x⊆∩{↑F ∣ F∈c(x)}=∩{↑y ∣ y≺σx}∩{↑F′ ∣ F′∈c(x)}⊆∩{↑y ∣ y≺σx}=↑x.

Thus ∩{↑*F*∣*F* ∈ *c*(*x*)} = ↑*x*. By Definition 19, *L* is countably *QC*-approximating.

By Lemma 16 and Proposition 20, we immediately have the following.

**Corollary 21 .**

For any poset *L*, the lattice *σ*_*c*_(*L*)^*op*^ is countably *QC*-approximating.

In the sequel, we explore relationships between countably *QC*-approximating lattices and GCD lattices.

**Proposition 22 .**

Every GCD lattice is weakly generalized countably approximating.

**Proof**

Let *L* be a GCD lattice. For all *x* ∈ *L* and *F* ∈ *P*_fin_(*L*), *F*⊲*x* implies *F*≪_*c*_ *x*. Then ↑*x*⊆∩{↑*F*∣*F* ∈ *ω*(*x*)}⊆∩{↑*F*∣*F* ∈ *P*_fin_(*L*) and *F*⊲*x*} = ↑*x*. So ↑*x* = ∩{↑*F*∣*F* ∈ *ω*(*x*)}. By Definition 10, *L* is weakly generalized countably approximating.

**Proposition 23 .**

Every GCD lattice is countably *QC*-approximating.

**Proof**

Let *L* be a GCD lattice. For each *x* ∈ *L* and *F* ∈ *P*_fin_(*L*), *F*⊲*x* implies *F*≺_*σ*_ *x*. Then ↑*x*⊆∩{↑*F*∣*F* ∈ *c*(*x*)}⊆∩{↑*F*∣*F* ∈ *P*_fin_(*L*) and *F*⊲*x*} = ↑*x*. Thus ↑*x* = ∩{↑*F*∣*F* ∈ *c*(*x*)}. By Definition 19, *L* is countably *QC*-approximating.

The following theorem characterizes GCD lattices.

**Theorem 24 .**

Let *L* be a complete lattice. Then the following statements are equivalent:

1. *L* is a GCD lattice;
2. *L* is countably *QC*-approximating and weakly generalized countably approximating.

**Proof**

(1)⇒(2): follows from Propositions 22 and 23.

(2)⇒(1): suppose that *L* is countably *QC*-approximating and weakly generalized countably approximating. Then for each *x* ∈ *L*, by the weakly generalized countably approximating property of *L*, we have ↑*x* = ∩{↑*F*∣*F* ∈ *ω*(*x*)}. Now for each *F* ∈ *ω*(*x*), we show that ↑*F* = ∩{↑*F*′∣*F*′ ∈ *P*_fin_(*L*) and *F*′≺_*σ*_ *F*}. To this end, it suffices to show that ∩{↑*F*′∣*F*′ ∈ *P*_fin_(*L*) and *F*′≺_*σ*_ *F*}⊆↑*F*. Suppose *t* ∈ ∩{↑*F*′∣*F*′ ∈ *P*_fin_(*L*) and *F*′≺_*σ*_ *F*} and *t* ∉ ↑*F*. Then for any *y*_*F*_ ∈ *F*, *t* ∉ ↑*y*_*F*_. By the countably *QC*-approximating property of *L*, there exists *F*_*y*_*F*__ ∈ *c*(*y*_*F*_) such that *F*_*y*_*F*__≺_*σ*_ *y*_*F*_ and *t* ∉ ↑*F*_*y*_*F*__. Let $\bar{F}={⋃}_{y_{F}\in F}F_{y_{F}}$. Then $\bar{F}$ is still finite and $\bar{F}{≺}_{\sigma }F$. It is clear that $t\notin ↑\bar{F}$, contradicting to that *t* ∈ ∩{↑*F*′∣*F*′ ∈ *P*_fin_(*L*) and *F*′≺_*σ*_ *F*}. Thus ↑*x* = ∩{↑*F*∣*F* ∈ *ω*(*x*)} = ∩{↑*F*′∣*F*′ ∈ *P*_fin_(*L*), ∃*F* ∈ *P*_fin_(*L*) such that *F*′≺_*σ*_ *F*≪_*c*_ *x*}.

Suppose *F*′≺_*σ*_ *F*≪_*c*_ *x*, we will show that *F*′⊲*x*. For any *A*⊆*L* with ∨*A* ≥ *x*, let *G* = {∨*E*∣*E* is a countable subset of *A*}. Then *G* is a countably directed set and ∨*G* = ∨*A* ∈ ↑*x*. Since *F*≪_*c*_ *x*, there exists a countable subset *E*⊆*A* such that ∨*E* = ∨↓*E* ∈ ↑*F*. By Remark 14 (1), ↓*E* is *σ*-Scott-closed. It follows from *F*′≺_*σ*_ *F* that ↓*E*∩↑*F*′ ≠ *∅*. This implies *A*∩↑*F*′ ≠ *∅*, showing that *F*′⊲*x*. Thus, ↑*x*⊆∩{↑*W*∣*W* ∈ *P*_fin_(*L*), *W*⊲*x*}⊆∩{↑*F*′∣*F*′ ∈ *P*_fin_(*L*), ∃ *F* ∈ *P*_fin_(*L*), *F*′≺_*σ*_ *F*≪_*c*_ *x*} = ↑*x*. So, ↑*x* = ∩{↑*W*∣*W* ∈ *P*_fin_(*L*) and *W*⊲*x*}. Therefore, *L* is a GCD lattice.

Recall that a poset *L* is called a* hypercontinuous poset* (see [13]) if for all *x* ∈ *L*, the set {*y* ∈ *L*∣*y*≺_*ν*(*L*)_ *x*} is directed and *x* = sup⁡{*y* ∈ *L*∣*y*≺_*ν*(*L*)_ *x*}, where *y*≺_*ν*(*L*)_ *x*⇔*x* ∈ int_*ν*(*L*)_↑*y*. A hypercontinuous poset which is also a complete lattice is called a* hypercontinuous lattice*.

**Lemma 25 (see [7], Theorem 4.1.4).**

Let *L* be a complete lattice. Then *L* is a GCD lattice if and only if *L* ^*op*^ is a hypercontinuous lattice.

It is easy to see that for a finite lattice *L*, both *L* and *L* ^*op*^ are continuous, and *ν*(*L*) = *σ*(*L*). It follows from ([14], Theorem 2.1) that *L* and *L* ^*op*^ are hypercontinuous lattices; hence by Lemma 25, *L* ^*op*^ and *L* are GCD lattices. By this observation, we see that every finite lattice is a countably *QC*-approximating lattice. So, countably *QC*-approximating lattices need not be distributive.

It is known from Proposition 4.1 in [12] that any countably *C*-approximating lattice is distributive. So, countably *QC*-approximating lattices need not be countably *C*-approximating.

**Lemma 26 (see [11], Theorem 3.4).**

Let *L* be a poset having countably directed joins. Then *L* is generalized countably approximating if and only if the lattice *σ*_*c*_(*L*) is hypercontinuous.

So, in view of Lemma 25, a poset having countably directed joins is generalized countably approximating if and only if the lattice *σ*_*c*_(*L*)^*op*^ is a GCD lattice. The following theorem gives comprehensive characterizations of generalized countably approximating posets.

**Theorem 27 .**

Let *L* be a poset having countably directed joins. Then the following statements are equivalent:

- *L* is a generalized countably approximating poset;
- *σ*_*c*_(*L*) is a hypercontinuous lattice;
- *σ*_*c*_(*L*)^*op*^ is a GCD lattice;
- *σ*_*c*_(*L*)^*op*^ is a weakly generalized countably approximating lattice.

**Proof**

(i)⇔(ii) by Lemma 26.

(ii)⇔(iii) by Lemma 25.

(iii)⇔(iv) follows from Theorem 24 and Corollary 21.
